# Correlation of *EYS* polymorphisms with lumbar disc herniation risk among Han Chinese population

**DOI:** 10.1002/mgg3.890

**Published:** 2019-07-30

**Authors:** Demin Ji, Wenhua Xing, Feng Li, Zhi Huang, Wenkai Zheng, Baoyang Hu, FangLin Niu, Yong Zhu, Xuejun Yang

**Affiliations:** ^1^ Inner Mongolia Medical University Hohhot China; ^2^ The Second Affiliated Hospital of Inner Mongolia Medical University Hohhot China; ^3^ Key Laboratory of Resource Biology and Biotechnology in Western China (Northwest University), Ministry of Education Xi'an China

**Keywords:** Case‐control study, *EYS*, Lumbar disc herniation (LDH), Single‐nucleotide polymorphism (SNP)

## Abstract

**Background:**

Lumbar disc herniation (LDH) is a common spinal disease in clinical practice. Once lumbar disc herniation occurs, it seriously reduces patient's quality of life. The *EYS* (eyes shut homolog) was discovered in recent years and it may be related to lumbar disc herniation. So we conducted a case–control study to explore the relationship between *EYS* polymorphism and lumbar disc herniation risk.

**Methods:**

We selected 5 single‐nucleotide polymorphisms (SNPs) of *EYS* gene in a case–control study with 508 cases and 508 healthy controls to evaluate the relatedness by using genetic model, haplotype, and stratification analysis.

**Results:**

We found that the minor alleles of rs62413038 (OR = 1.21, 95%CI: 1.01–1.43, *p* = .036) and rs9450607 (OR = 1.26, 95% CI: 1.05–1.53, *p* = .016) were associated with an increased risk of lumbar disc herniation in the allelic model analysis. In the genotypic model analysis, rs62413038 displayed a significantly increased risk of lumbar disc herniation in log‐additive models (OR = 1.20, 95% CI: 1.01–1.43, *p* = .039). While the rs9450607 was also obviously associated with an increased lumbar disc herniation risk in recessive (OR = 1.98, 95% CI: 1.24–3.13, *p* = .004) and log‐additive models (OR = 1.27, 95% CI: 1.05–1.55, *p* = .014). In addition, in the haplotype analyses of the SNPs, we found that the “CGGA” haplotype of rs1482456, rs9342097, rs9450607, and rs7757884 was associated with lumbar disc herniation. (OR = 0.52, 95% CI: 0.30–0.89, *p* = .017).

**Conclusion:**

These results suggest that *EYS* polymorphism may be associated with lumbar disc herniation among Han Chinese population. It also opens up a new exploration direction for the etiology of lumbar disc herniation.

## 1 INTRODUCTION

Lumber disc herniation (LDH) is a common degenerative disease of the lumbar spine, and is the main leading cause of spinal surgery in adults. Although it is not a malignant condition, it is considered a global health problem. It leads to chronic low back pain, negative impacts on physical and work activities, decreased quality of life, and psychological distress in affected individuals (Casa et al., 2016). Generally, LDH is a complex disease involving multifactorial interactions. In terms of current research, trauma, environmental and genetic factors are considered to be the main causes of disease. However the precise etiology and pathogenesis underlying of LDH are complex and still poorly understood (Mu, Ge, Zuo, Chen, & Huang, 2014).

Recently, it was suggested that the heritability of LDH is 52% to 68%, indicating that genetic factors might play a decisive role in the pathogenesis of LDH (MacGregor, Andrew, Sambrook, & Spector, 2004). To date, with the completion of high‐quality sequencing of the human genome and a deeper understanding of disease mechanisms at the cellular and molecular level, the genes responsible for susceptibility to many complex diseases, including LDH, have been identified (Guo, Liu, Zhang, Guo, & Wu, 2011; Zhang et al., 2010; Zhang, Sun, Liu, & Guo, 2008). Considering that LDH is a frequent disorder among adults, understanding its relationship to genetic factors can provide new prevention and intervention measures for individuals affected by the disease. With the identification of the polymorphisms of various LDH‐related genes, we lay a foundation for elucidating the genetics of intervertebral disc herniation and also open a new door for us to study the etiology of LDH (Casa et al., 2016).

*EYS* is a new gene identified at the RP25 locus on chromosome 6q12 commonly mutated in autosomal recessive retinitis pigmentosa (Abd El‐Aziz et al., 2008; Zelhof, Hardy, Becker, & Zuker, 2006). According to relevant research in an unselected birth cohort, they found locus 6q12 which the *EYS* located in was associated with nitric oxide levels. Moreover, we know that disc cells were able to release nitric oxide and nitric oxide might play an important role in the pathogenesis of disc degeneration through the induction of apoptosis of disc cells in situ (Fuchs et al., 2017, 2011; Kohyama, Saura, Doita, & Mizuno, 2000). Therefore, we hypothesize that mutations in the *EYS* (located in chromosome 6q12) cause problems in the regulation mechanism of the *EYS* in the intervertebral disc cells, leading to abnormal nitric oxide metabolism, followed by apoptosis of the intervertebral disc cells, which eventually leads to degeneration and herniation of the intervertebral disc (Janeczko, Janeczko, Chrzanowski, & Zielinski, 2014; Videman et al., 2009).

Therefore, we infer that *EYS* polymorphism may affect individual LDH risk. We conducted a case–control study to evaluate the possible association of *EYS* polymorphisms at allele, genotype, and haplotype interface with the development of LDH among Han Chinese population and the results of this study will further confirm the role of *EYS* in the pathogenesis of LDH.

## 2 MATERIALS AND METHODS

### 2.1 Study participants

A case–control study involving of 508 LDH patients and 508 controls was conducted at The Second Affiliated Hospital of Inner Mongolia Medical University, Inner Mongolia, China. All cases were diagnosed with LDH according to the latest clinical guidelines. The inclusion criteria for cases group were: patients who had typical clinical symptoms and physical signs, patients' whose imaging examination showed prominent intervertebral disc. LDH symptoms were those described as follows:(a) lower back pain, (b) pain in the inferior lumbar part of the spine and regional typical sciatica; (c) difficulty in straight leg raising test and augmentation test; (d) the limited lumbar flexion range. Lumbar spine MRI confirmed the patients with LDH according to the Pfirrmann grading system. Patients with complicated blood diseases, autoimmune diseases, tumors, trauma, rheumatoid arthritis, and related lumbar spine disease containing lumbar spinal stenosis, spinal congenital dysplasia, intraspinal tumor, and spondylolisthesis were excluded from this study. The control subjects were recruited from the health checkup center of The Second Affiliated Hospital of Inner Mongolia Medical University, which they had visited for an annual health examination. These volunteers were not scanned by MRI and no history of sciatica and low back pain. Inclusion criteria of the control group were: (a) individuals had no family history of LDH; (b) individuals without spinal instability from trauma, scoliosis, spondylolisthesis, osteoarthritis, rheumatism and rheumatoid arthritis; (c) individuals without infections and any history of tumors. All studied individuals were Chinese Han subjects tracing back at least three generations. Demographic and related clinical data of the study population were collected by a face‐to‐face questionnaire and medical case record search. All of the participants were genetically unrelated ethnic Han Chinese from Inner Mongolia and provided written informed consent for their participation in the present study.

### 2.2 Ethical compliance

This study was approved by the ethics committee of The Second Affiliated Hospital of Inner Mongolia Medical University. All subjects provided written informed consent before the collection of blood samples.

### 2.3 DNA isolation

We used venipuncture blood vacutainer collection tubes to collect peripheral blood samples from each subject and then stored at −80°C for further use. We used the GoldMag‐Mini Whole Blood Genomic DNA Purification Kit (GoldMag. Co. Ltd.) to extract genomic DNA from blood samples following the manufacturer's instructions. We assessed the purity and concentration of the extracted DNA using a spectrophotometer (NanoDrop 2000; Thermo Fisher Scientific) by absorbance measurements at 260 and 280 nm.

### 2.4 SNP selection and genotyping

Five SNPs from *EYS* were chosen for analysis in this study and they were selected from the GWAS, NCBI dbSNP (http://www.ncbi.nlm.nih.gov/snp) and the 1000 Genomes Project databases (http://www.internationalgenome.org/). All five SNPs had minor allele frequencies >5% in Han Chinese Beijing population. We used the Agena Bioscience Assay Design Suite V2.0 software (https://agenacx.com/online-tools/) to design the primers of PCR amplification and extension of the five selected SNPs. These SNPs in *EYS* were genotyped in the case and control groups using the Agena MassARRAY platform with iPLEX gold chemistry (Agena Bioscience, San Diego, CA) according to the manufacturer's instructions. We used the Agena Bioscience TYPER software (version 4.0) to manage and analyze data.

### 2.5 Statistical analysis

Data were analyzed using SPSS version 18.0 statistical software (SPSS Inc, Chicago, IL, United States). Allele frequency of each SNP in the control subjects was analyzed using χ^2^ test to evaluate departure from Hardy–Weinberg equilibrium (HWE). The genotype and allele distributions for SNPs in LDH patients and control subjects were compared by χ^2^ test. Under four different genetic models (codominant, dominant, recessive, and log‐additive), we assessed the association between each SNP and the risk of LDH while adjusting for age and gender. Next, using PLINK software, version 1.07 (http://zzz.bwh.harvard.edu/plink/ld.shtml), association intensity between each genotype and steroid‐induced ONFH risk was estimated by odd ratios (OR) and the corresponding 95% confidence intervals (CIs) under four genetic models. All two‐sided *p* values < .05 were considered statistically significant. Associations between SNPs and risk of LDH were tested in genetic models using SNP Stats software. The SHEsis software platform was used for the analyses of linkage disequilibrium and haplotype construction.

## 3 RESULTS

Table 1 shows the volunteers' characteristics. A total of 508 cases of LDH (297 males and 211 females, mean ± Standard Deviation: 49.16 ± 14.90) and 508 healthy controls (297 males and 211 females, mean ± Standard Deviation: 48.49 ± 13.71) were enrolled in our study. There were no statistically significant differences on the gender and age distribution between the case and control groups.

**Table 1:** Characteristics of cases and controls in this study

Variable(s)	Case (*n* = 508)	Control (*n* = 508)	*p* value
Sex *N*(%)			1.000^a^
Male	297 (58.5%)	297 (58.5%)	
Female	211 (41.5%)	211 (41.5%)	
Age, year (mean ± *SD*)	49.16 ± 14.90	48.49 ± 13.71	.457^b^

a Two‐sided Chi‐squared test.

b Independent samples *t* test.

Five SNPs were selected for our study which included rs62413038, rs1482456, rs9342097, rs9450607, and rs7757884. The basic information of the SNPs including chromosomal position, minor allele frequency (MAF) of cases and controls, and Hardy–Weinberg equilibrium (HWE) test results are summarized in Table 2. All five SNPs were in Hardy–Weinberg equilibrium. (*p* > .05) (Table 2).We compared the differences in frequency distributions of alleles between cases and controls by χ^2^ test and found that two significant SNPs in *EYS* were associated with LDH risk (rs62413038 OR = 1.21; 95% CI 1.01–1.43; *p* = .036, rs9450607 OR = 1.26; 95% CI 1.05–1.53; *p* = .016).

**Table 2:** Allele frequencies in cases and controls and OR estimates of LDH

SNP	Gene	Chromosome	Alleles A/B	MAF		*p* a value for HWE	OR	Allele model	
				case	control			95% CI	*p* b
rs62413038	*EYS*	6	G/T	0.473	0.427	1	1.21	1.01–1.43	.036
rs1482456	*EYS*	6	A/G	0.405	0.374	.636	1.14	0.95–1.36	.158
rs9342097	*EYS*	6	G/T	0.346	0.311	.351	1.18	0.98–1.41	.088
rs9450607	*EYS*	6	A/G	0.325	0.276	.06	1.26	1.05–1.53	.016
rs7757884	*EYS*	6	A/C	0.286	0.252	.1	1.19	0.98–1.45	.08

Note

*p* < .05 indicates statistical significance.

Abbreviations: 95% CI, 95% confidence interval; HWE, Hardy–Weinberg equilibrium; MAF, minor allele frequency; SNP, single‐nucleotide polymorphism; OR, odds ratio.

a *p* was calculated by exact test.

b *p* was calculated by Pearson Chi‐squared test.

Then we assumed that the minor allele of each SNP was a risk allele compared to the wild type allele and four multiple inheritance models (codominant, dominant, recessive, and log‐additive models) were applied for analyzing the association between SNPs and LDH risk by logistic regression analysis adjusted for age and gender. (Table 3) In the genetic model analyses, the genotype G/T of rs62413038 was associated with an increased risk of LDH under the log‐additive model (OR = 1.20, 95% CI: 0.94–1.61, *p* = .039). The genotype G/G‐G/A of rs9450607 was also significantly associated with an increased LDH risk in recessive model (OR = 1.98, 95% CI: 1.24–3.13, *p* = .004) and log‐additive model (OR = 1.27, 95% CI: 1.05–1.55, *p* = .014).

**Table 3:** Genotypic model analysis of the relationship between SNPs and the risk of LDH

SNP ID	Model		Genotype	Case	Control	OR (95% CI)	*p*‐value
rs62413038	Codominant		T/T	144	167	1	
			G/T	247	248	1.15 (0.87–1.53)	.036*
			G/G	117	93	1.46 (1.03–2.07)	.126
	Dominant		T/T	144	167	1	
			G/G‐G/T	364	341	1.24 (0.94–1.61)	.062
	Recessive		G/G‐G/T	391	415	1	
			G/G	177	93	1.34 (0.99–1.82)	
	Log‐additive		–	–	–	1.20 (1.01–1.43)	.039*
rs9450607	Codominant		A/A	234	258	1	
			G/A	218	220	1.09 (0.84–1.41)	.003*
			G/G	56	30	2.06 (1.28–3.32)	
	Dominant		A/A	234	258	1	
			G/G‐G/A	274	250	1.21 (0.94–1.55)	.132
	Recessive		G/G‐G/A	452	478	1	
			G/G	56	30	1.98 (1.24–3.13)	.004*
	Log‐additive		–	–	–	1.27 (1.05–1.55)	.014*

Note

* *p* < .05 indicates statistical significance.

Abbreviations: OR, odds ratio; 95% CI, 95% confidence interval.

We still analyzed the effect of these SNPs and LDH association by age and gender stratification in Table 4. We found that only one SNP polymorphism was associated with LDH in age ≥ 49 population (rs9450607, OR = 2.18, 95% CI: 1.09–4.35, *p* = .028), meanwhile for age < 49, no more SNPs were found significant. For male, we found that three SNPs were associated with LDH (rs62413038, OR = 1.77, 95% CI: 1.12–2.79, *p* = .014; rs9342097, OR = 1.94, 95% CI: 1.11–3.37, *p* = .020; rs9450607, OR = 2.10, 95% CI: 1.13–3.88, *p* = .019), however for female population, no SNPs were found.

**Table 4:** The association between SNPs and age, gender analysis of LDH patients

SNP	Gene	Allele	<49			≥49			Male			Female		
			OR	95% CI	*p* #	OR	95% CI	*p* #	OR	95% CI	*p* *	OR	95% CI	*p* *
rs62413038	*EYS*	G/T	1.48	0.90–2.42	.121	1.37	0.83–2.28	.22	1.77	1.12–2.79	.014	1.09	0.63–1.91	.752
rs1482456	*EYS*	C/T	1.39	0.81–2.38	.228	1.2	0.70–2.07	.505	1.56	0.95–2.56	.079	1.03	0.57–1.87	.923
rs9342097	*EYS*	G/T	1.48	0.82–2.66	.19	1.63	0.88–3.03	.12	1.94	1.11–3.37	.020	1.23	0.64–2.37	.543
rs9450607	*EYS*	G/A	1.88	0.97–3.66	.062	2.18	1.09–4.35	.028	2.1	1.13–3.88	.019	2.01	0.94–4.26	.071
rs7757884	*EYS*	C/A	1.89	0.92–3.88	.085	1.68	0.78–3.62	.183	1.87	0.95–3.67	.068	1.7	0.74–3.92	.211

Note

*p* < .05 indicates statistical significance.

Abbreviations 95% CI, 95% confidence interval; OR, odds ratio.

# *p* values were calculated with Pearson's Chi‐squared test adjusted by gender.

* *p *values were calculated with Pearson's Chi‐squared test adjusted by age.

**Figure 1:**
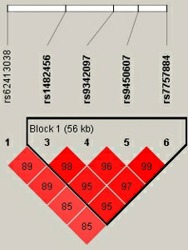
Linkage disequilibrium (LD) analysis of the association between all the SNPs of the *EYS* and lumbar disc herniation (LDH)

Finally, we studied the linkage disequilibrium (LD) and haplotypes analyses of SNPs (Figure 1, Table 5). The LD block included rs1482456, rs9342097, rs9450607, and rs7757884, then, we found that the “CGGA” haplotype of rs1482456, rs9342097, rs9450607, and rs7757884 was associated with LDH risk. (OR = 0.52, 95% CI: 0.30–0.89, *p* = .017).

**Table 5:** *EYS * haplotype frequencies and their associations with LDH risk

rs1482456	rs9342097	rs9450607	rs7757884	Freq	OR(95％ CI)	*p* *	OR(95％ CI)	*p* #
C	G	G	C	0.7490	0.89 (0.73–1.10)	.288	0.89 (0.73–1.10)	.273
C	G	G	A	0.9792	0.51 (0.30–0.88)	.014	0.52 (0.30–0.89)	.017
C	G	A	A	0.9625	1.37 (0.83–2.56)	.211	1.37 (0.83–2.25)	.216
C	T	A	A	0.9358	1.02 (0.71–1.46)	.928	1.02 (0.71–1.47)	.916
T	T	A	A	0.6235	0.84 (0.70–1.01)	.069	0.84 (0.70–1.01)	.066

Note

*p* < .05 indicates statistical significance.

Abbreviations: 95% CI, 95% confidence interval; OR, odds ratio.

* *p* was calculated by logistic regression.

# *p* was calculated by unconditional logistic regression adjusted for age and gender.

## 4 DISCUSSION

As time goes, the idea that lumbar disc herniation was affected by genetic factors had been accepted by more and more people (Mayer et al., 2013). In the present case–control study, we investigated the associations between five SNPs of the *EYS* gene and the risk of LDH. We demonstrated that *EYS* genetic polymorphisms (rs62413038, rs9342097 and rs9450607) were associated with an increased risk of LDH among Han Chinese population. We found that sex and age differences may interact with *EYS* polymorphisms to affect the development of LDH. We also found that the “CGGA” haplotype of *EYS* was related to a 0.52‐fold decrease in the risk of LDH.

In 2013, a meta‐analysis of 4,600 subjects was performed based on new genetic variations associated with lumbar disc degeneration in Northern Europe. In this multivariate analysis, the locus rs11754641 on *EYS* was also included for association analysis. The final conclusion was that this site was associated with lumbar disc degeneration (Williams et al., 2013). From our research, we could observe that rs9450607 in *EYS* was found by the association with increased LDH risk and it was more significant among people over 49 years old. In addition, it also increased 2.10‐fold risk of LDH in males compared to females. This result demonstrated that rs9450607 of *EYS* was associated with the susceptibility to LDH in Han Chinese population and also the risk association of the polymorphisms which was gender‐dependent.

Recently, Isackson et al indicated that the sequence similarity of the EGF‐like repeats in the N‐terminal region of the expression products of *EYS* in human eyes to the EGF‐like repeats of Notch1. Certainly, proteins containing similar clusters of EGF‐like domains in the Notch pathway have been shown to bind to each other. Thereby, it suggested that the expression products of *EYS* may play a role in the Notch signaling pathway (Isackson et al., 2011). Meanwhile Wang et al also found an increase in Notch signaling protein expression in human degenerated intervertebral discs, including the expression of Notch receptor and target genes in degenerated intervertebral discs. Coincidentally, they compared degenerated discs with nondegenerative disc samples and found increased Notch2 receptor expression in the nucleus pulposus of degenerated discs in middle age (Wang et al., 2013). From the above studies, we could find that *EYS* products may be related to disc degeneration or herniation through Notch pathway. Therefore, in combination with our experimental results, we propose for the first time that *EYS* polymorphism was associated with the risk of lumbar disc herniation in Han Chinese population (Risbud & Shapiro, 2014).

Although the results of our present study provide scientific evidence about *EYS* and LDH in the future studies, our study has some limitations. Firstly, the subjects of investigation were enrolled from the identical hospital and the control group did not undergo MRI examination. Therefore, selection biases could not rule out. Secondly, some potential confounding factors such as occupational exposures and physical activity were not included in our analysis and should be assessed in the future. Thirdly, our study does not include an analysis of biological functions yet, which will be crucial for elucidating the role of *EYS* in LDH (Liu et al., 2016; Zhu et al., 2018).

## 5 CONCLUSION

In summary, we have confirmed for the first time that the association three SNPs (rs62413038, rs9342097, and rs9450607) of *EYS* gene with increased risk of LDH among Han Chinese population. While larger population‐based studies and further functional researches are needed to confirm our results.
